# When Bioengineering Met Artificial Intelligence: From Machine Learning to Intelligent Bioengineering Systems

**DOI:** 10.3390/bioengineering13080928

**Published:** 2026-08-16

**Authors:** Daniele Giansanti

**Affiliations:** Centro IATIS, Istituto Superiore di Sanità, 00161 Roma, Italy; daniele.giansanti@iss.it; Tel.: +39-06-49902701

Artificial intelligence and bioengineering have developed along different historical trajectories, yet their areas of interest have progressively converged. Bioengineering has traditionally combined engineering principles, biological systems, and technological innovation to address complex biological and medical problems, while artificial intelligence has evolved through successive generations of computational approaches aimed at learning, recognizing patterns, reasoning, predicting, and supporting intelligent decision-making. Their convergence is therefore not the result of a single technological development, but of a gradual process in which increasingly sophisticated computational approaches have become embedded within bioengineering research and applications.

This convergence is particularly relevant today, as AI is moving beyond its traditional role as a tool for analyzing bioengineering data and now plays an increasingly prominent role in the design, prediction, optimization, control, and adaptation of bioengineered systems. At the same time, new developments—including machine learning, deep learning, generative AI, foundation models, and digital twins—are expanding the range of possible interactions between computational intelligence and biological systems.

In this Editorial, we briefly trace this evolution from a historical and conceptual perspective, map some representative milestones in the convergence of the two fields using PubMed, and then consider selected areas in which AI is currently shaping bioengineering research. Finally, we look at emerging directions that may define the next phase of this convergence, providing context for the Special Issue “Artificial Intelligence in Bioengineering: Innovations, Challenges, and Future Directions.”

*Defining the Two Domains*

Merriam-Webster defines bioengineering as “the application of engineering principles, practices, and technologies to the fields of medicine and biology, especially in solving problems and improving care (as in the design of medical devices and diagnostic equipment or the creation of biomaterials and pharmaceuticals)” [1]. This definition emphasizes the inherently interdisciplinary nature of bioengineering, in which engineering concepts and technologies are applied to biological and medical systems to address complex problems, develop innovative solutions, and improve the prevention, diagnosis, treatment, monitoring, and rehabilitation of human conditions. Importantly, the definition is not restricted to a specific technological domain. Bioengineering may encompass, among other areas, biomedical devices and instrumentation, biosensors, physiological signal analysis, medical imaging, biomaterials, tissue engineering, prostheses, robotics, rehabilitation technologies, computational modeling, and other technologies operating at the interface between engineering and biological systems. The breadth of this definition is particularly relevant when considering the increasingly prominent role computational technologies play in contemporary bioengineering, where biological information can be acquired, processed, modeled, interpreted, and translated into adaptive technological solutions.

In the same dictionary, artificial intelligence (AI) is defined as “the capability of computer systems or algorithms to imitate intelligent human behavior” [2]. Although concise, this definition captures a fundamental characteristic of AI: the development of computational systems capable of performing functions that traditionally require different forms of human intelligence. These functions may include learning from data, recognizing patterns, reasoning, solving problems, predicting outcomes, interpreting complex information, generating content, and supporting or making decisions. In this context, AI is an umbrella concept encompassing a broad and evolving family of computational approaches rather than a single technology or algorithm. This broader landscape includes, among others, machine learning, artificial neural networks, deep learning, expert systems, reinforcement learning, and, more recently, generative AI and foundation models [3].

The relationship between these approaches has also evolved over time. Machine learning has become one of the main approaches through which AI systems acquire knowledge or improve their performance from data, while artificial neural networks represent a family of computational models that have played a central role in the development of modern machine learning and deep learning. Deep learning, in turn, has substantially expanded the ability of AI systems to learn complex representations from large and heterogeneous datasets. More recently, generative AI and foundation models have further broadened the scope of AI by enabling systems to generate, transform, and integrate complex forms of information across different modalities. These developments illustrate that the terminology associated with AI is dynamic and reflects successive stages of technological and methodological evolution [3].

The conceptual relationship between bioengineering and AI therefore becomes particularly relevant at their intersection. Bioengineering provides the biological, physiological, technological, and clinical contexts in which complex problems must be understood and addressed, whereas AI provides computational approaches capable of processing increasingly large and heterogeneous datasets and supporting functions such as recognition, prediction, modeling, optimization, and decision-making. Their convergence can consequently occur at multiple levels, ranging from the analysis of biological signals and images to the design of biomaterials, the control of robotic and assistive systems, the development of personalized interventions, and the modeling and simulation of complex biological processes.

Importantly, these two fields have not evolved independently of computational developments. The incorporation of computational methods into bioengineering preceded the widespread adoption of the explicit term “artificial intelligence.” Earlier studies may have described approaches in terms such as pattern recognition, automated classification, expert systems, neural networks, or other forms of computational analysis, even when the term AI was not explicitly used. Some of these approaches subsequently became integral components of the broader AI landscape, while others evolved along partially independent trajectories. Consequently, a historical analysis of the relationship between AI and bioengineering cannot rely exclusively on the appearance of the phrase “artificial intelligence” in the scientific literature.

The historical convergence of these fields should therefore be understood not simply as the moment when the term “artificial intelligence” first appeared in bioengineering, but as a progressive process in which increasingly sophisticated computational approaches became integrated into the study, design, monitoring, modeling, and control of biological and bioengineered systems. This perspective is particularly important when examining the evolution of the scientific literature, because different generations of technologies have been described using different terminology. Accordingly, when tracing the historical relationship between AI and bioengineering, it is useful to consider both the explicit term “artificial intelligence” and related AI approaches that may have appeared earlier in the literature. Such an approach allows the evolution of the field to be interpreted as a continuum, extending from early computational and pattern-recognition approaches to machine learning, deep learning, and, more recently, generative and foundation model-based systems.

*A Historical Map of Convergence*

To provide a simple historical map of the convergence between bioengineering and AI, we explored PubMed qualitatively using Title/Abstract (tiab) searches (Table 1). Different keyword combinations were used to highlight distinct stages of this convergence: *bioengineering* alone to identify the emergence of the field; *artificial intelligence* alone to mark the emergence of AI as an explicit concept; and *bioengineering* combined with AI and its main related approaches and components, including machine learning, artificial neural networks, deep learning, expert systems, pattern recognition, and other computational approaches, to capture earlier and evolving forms of AI-related activity in bioengineering. Finally, *bioengineering* combined specifically with “*artificial intelligence*” was used to identify records in which the two concepts were explicitly connected. The resulting publications are presented simply as historical milestones illustrating the progressive convergence of the two fields, rather than as the results of a systematic literature review.

To provide a simple historical map of the relationship between bioengineering and AI, we explored the PubMed-indexed literature qualitatively using Title/Abstract (tiab) searches (Table 1). The different search positions were used to highlight distinct stages in the evolution of the two fields rather than to identify all relevant publications. Position 1 focused on broad AI-related terms and concepts, Position 2 on bioengineering alone, Position 3 on the explicit term artificial intelligence, Position 4 on the combination of bioengineering with broader AI-related terms and approaches, and Position 5 on the explicit combination of bioengineering and artificial intelligence. 

The publications identified through these searches are therefore presented as representative historical milestones, rather than as the results of a systematic literature review. An interesting starting point emerges from the broader AI-related terminology (Table 1, Position 1) The earliest record retrieved through the broad AI-related terminology search search dates back to 1879 [4], with “*The Medical Expert System in Germany*”. Although the historical terminology and meaning of “expert system” clearly differ from those used in the context of modern AI, the record provides an intriguing early historical record containing terminology that can be viewed in relation to later expert-system approaches within the broader AI landscape.

This record is not interpreted as evidence of AI in the modern sense, but simply as an early example of terminology or concepts that, from a broader historical perspective, can be connected to the subsequent development of computational intelligence. This finding illustrates an important point for the historical mapping: the intellectual roots of AI-related concepts may precede the terminology that was later adopted to define the field itself.

The trajectory of bioengineering can be traced explicitly to the 1950s. One of the earliest PubMed-indexed records identified through the *bioengineering*-only search (Table 1, Position 2) is Thorndike’s “*Bioengineering—Blueprint for Progress*”, published in 1954 [5]. This early contribution is particularly relevant because it explicitly uses the term *bioengineering* and reflects the emerging view of engineering as a means of addressing biological and medical problems. The context of artificial limbs is also historically meaningful: the human body was frequently approached as a complex biological system in which engineering principles could be applied to restore or enhance function. At this stage, however, the field was fundamentally technological and biological rather than computational.

A parallel trajectory is evident in the development of artificial intelligence as an explicit scientific concept. Maron’s “*Artificial Intelligence and Brain Mechanisms*”, published in 1963 [6], represents an early PubMed-indexed record identified through the AI-only search (Table 1, Position 3). Its importance for the present historical map lies primarily in the explicit use of the expression *artificial intelligence*. At this point, AI was emerging as a recognizable computational field concerned with the relationship between intelligent behavior, information processing, and computational representations. Bioengineering and AI had therefore developed along initially distinct trajectories: one centered on the application of engineering principles to biological systems, and the other on the development of computational approaches capable of reproducing or supporting aspects of intelligent behavior.

The next stage is particularly important for understanding how these two trajectories began to approach one another. By the early 1980s, bioengineering research oftenincorporated computational methods capable of analyzing complex biological information. A representative example is provided by Moore and Amplatz’s 1981 study, “*Left ventriculometry: the Minnesota experience with a bioengineering approach*” [7], identified through the broader AI-related search (Table 1, Position 4). The study describes computerized analysis and an automatic pattern-recognition method for evaluating ventricular wall motion. Although *pattern recognition* should not be considered synonymous with artificial intelligence, it represents an important computational approach within the broader historical development of AI-related methods. Its presence in a bioengineering application illustrates an important transition from engineering systems that primarily measured or manipulated biological phenomena toward systems that could also computationally interpret biological information.

This intermediate phase is particularly relevant because it shows that the convergence of bioengineering and AI did not necessarily begin when researchers started explicitly using the words *artificial intelligence*. Computational methods such as pattern recognition, artificial neural networks, expert systems, automated classification, and related approaches emerged at different times and under different terminologies. Some subsequently became central components of the AI landscape, while others evolved along partially independent computational trajectories. The historical record therefore suggests a gradual incorporation of increasingly sophisticated computational capabilities into bioengineering rather than a single point of technological convergence.

A further milestone is reached when the connection becomes explicitly expressed through the terminology of both fields. Sacha and Varona’s 2013 paper, “*Artificial intelligence in nanotechnology*” [8], represents an example identified through the search combining bioengineering-related terminology with ‘artificial intelligence (Table 1, Position 5). This search is deliberately more restrictive than Position 3: rather than capturing computational approaches that may be historically related to AI, it identifies records in which *artificial intelligence* is explicitly connected with the bioengineering-related domain in the Title/Abstract search strategy. Although the 2013 contribution focuses primarily on nanotechnology, it explicitly discusses bioengineering among the areas that may benefit from AI, illustrating a period in which AI was increasingly recognized as a technological paradigm with relevance across bioengineering-related domains.

The sequence identified through the qualitative PubMed Title/Abstract searches described above provides a historical perspective on the relationship between the two fields.

The sequence therefore illustrates a progressive conceptual transition. Position 1 reaches furthest back in time, showing that AI-related concepts and approaches have historical roots predating the explicit terminology of AI. Position 2 identifies the emergence of bioengineering as a defined field, while Position 3 marks the emergence of artificial intelligence as an explicit scientific concept. The subsequent appearance of computational approaches within bioengineering (Position 4), exemplified by automatic pattern recognition, provides an intermediate bridge between the two trajectories. Finally, Position 5 captures the stage at which bioengineering and artificial intelligence become explicitly connected in the Title/Abstract of scientific publications.

Taken together, these milestones suggest that the history of AI in bioengineering is better understood as a progressive convergence rather than a single historical event. Bioengineering first developed as an interdisciplinary application of engineering to biological and medical problems; AI emerged as a distinct computational paradigm; computational approaches associated with the broader AI landscape were progressively incorporated into bioengineering; and, eventually, artificial intelligence itself became explicitly recognized as a relevant technological dimension of bioengineering. The subsequent evolution from machine learning and deep learning to generative AI and foundation models represents a further acceleration of this convergence, with computational intelligence increasingly becoming an integral component of the design, analysis, modeling, optimization, and control of bioengineered systems.

*Where Bioengineering Meets AI Today*

The historical convergence described above has now developed into a broad and rapidly expanding research landscape. AI is being incorporated into bioengineering not simply as a tool for data analysis, but as a means of prediction, optimization, design, automation, and adaptive control. Current areas of particular interest include tissue engineering and biomaterials, biofabrication and 3D bioprinting, biosensing and wearable systems, robotics and rehabilitation, and prosthetic and orthotic technologies.

One major area of interest is tissue engineering and biomaterials, in which machine learning is being explored for biomaterial design, scaffold fabrication, tissue regeneration, and the prediction of biological and mechanical properties. Recent reviews specifically describe the growing integration of machine learning and AI across these stages, suggesting a transition from conventional trial-and-error approaches toward increasingly data-driven bioengineering design [9,10].

Closely related to this is the emergence of AI-enabled biofabrication and 3D bioprinting. Machine and deep learning have been applied to bioink selection, printing parameters, process optimization, image-based monitoring, defect detection, and prediction of the biological and mechanical properties of bioprinted constructs [11].

A second important frontier concerns intelligent biosensing and wearable bioengineering systems. AI is often combined with biosensors and wearable devices to interpret physiological signals, support continuous monitoring, detect patterns, and enable more personalized and adaptive systems. Recent systematic evidence demonstrates the growing use of AI with wearable biosensors and smart health devices, while also highlighting challenges related to data heterogeneity, limited datasets, generalizability, and real-world implementation [12,13].

The convergence is particularly evident in robotics, prostheses, orthoses, and rehabilitation technologies. Systematic reviews have documented the use of AI and machine learning for movement classification, trajectory prediction, patient assessment, robotic control, and personalization of rehabilitation interventions [14,15]. In parallel, machine learning has been investigated in prosthetic and orthotic systems for movement control, prosthesis selection, training, and other forms of functional adaptation [16].

Across these domains, a common transformation can be observed: AI is progressively moving from being an analytical layer applied to bioengineering data toward becoming an active component of bioengineering systems themselves. The emerging paradigm is therefore not simply AI applied *to* bioengineering, but increasingly intelligent bioengineering, in which sensing, modeling, prediction, optimization, control, and adaptation are integrated into the design and operation of biological and engineered systems.

This transition also brings important challenges. The increasing complexity of AI-enabled bioengineering systems raises questions concerning data quality, model generalizability, interpretability, reproducibility, validation, and real-world robustness. These challenges are particularly relevant when AI moves from retrospective data analysis toward real-time prediction, adaptive control, or autonomous decision-making. The systematic literature in robotics, rehabilitation, wearable systems, and other emerging areas illustrates that the future development of intelligent bioengineering will depend not only on improving algorithmic performance, but also on demonstrating reliability, generalizability, and effective integration within complex biological and engineered environments.

*Emerging Hot Topics and Future Directions*

Beyond these established areas, several emerging directions are further expanding the boundaries of AI-driven bioengineering. Digital twins represent one particularly promising frontier, combining computational modeling, data acquisition, simulation, and AI-enabled approaches to create dynamic representations of biological or bioengineered systems [17,18]. Recent reviews have highlighted their growing application in precision health and patient-specific modeling, while also emphasizing challenges related to validation, interoperability, standardization, and real-world implementation.

Another rapidly developing direction is AI-assisted biomaterial discovery and design, in which AI is frequently used not only to predict material properties but also to guide material generation, fabrication, and optimization [19,20]. This represents an important conceptual evolution, as AI moves from being primarily an analytical tool applied to existing bioengineering systems toward becoming an active instrument for designing new biological and engineered materials.

These developments are accompanied by growing interest in multimodal AI, foundation models, intelligent sensing, closed-loop systems, and increasingly autonomous bioengineering platforms. Multimodal approaches may enable the integration of heterogeneous biological, physiological, imaging, and engineering information, while generative AI and foundation models may provide new tools for knowledge integration, design, simulation, and interaction with complex bioengineering datasets. Similarly, closed-loop and adaptive systems may increasingly combine sensing, AI-based interpretation, prediction, and control within a single bioengineering framework.

Together, these emerging directions suggest a progressive shift from AI-supported bioengineering toward predictive, adaptive, and increasingly intelligent bioengineering systems. The central question is therefore no longer simply whether AI can be applied to bioengineering, but how increasingly capable computational systems can become integrated into the design, operation, optimization, and adaptation of bioengineered systems.

*A Special Issue at the Meeting Point of Artificial Intelligence and Bioengineering: Innovations, Challenges, and Future Directions.*

Against this rapidly evolving background, the Special Issue “Artificial Intelligence in Bioengineering: Innovations, Challenges, and Future Directions” [21] provides an opportunity to bring together the different dimensions of this evolving relationship. From the historical emergence of bioengineering and artificial intelligence as distinct fields, through their progressive convergence, to the current integration of AI into tissue engineering, biomaterials, biofabrication, biosensing, wearable systems, robotics, rehabilitation, prosthetic and orthotic technologies, and the more recent development of digital twins, AI-driven material design, multimodal approaches, and increasingly adaptive and intelligent systems, the landscape of AI in bioengineering is becoming progressively broader and more interconnected. The contributions to this Special Issue provide a timely opportunity to examine this evolution from different perspectives, capturing both established applications and emerging directions, as well as the methodological and translational challenges that accompany them. In this sense, the Special Issue offers a snapshot of a field that is moving rapidly from the application of AI to bioengineering toward the development of bioengineering systems that are designed, informed, and operated by artificial intelligence.

## Figures and Tables

**Table 1:** Used composite keys.

Position	Brief Description	PubMed Title/Abstract Search
1	Broad AI-related landscape—identifies the earliest records containing AI-related terms and approaches in tiab, including expert systems, pattern recognition, neural networks, machine learning, and more recent AI technologies.	(“artificial intelligence” [Title/Abstract] OR “AI” [Title/Abstract] OR “machine learning” [Title/Abstract] OR “deep learning” [Title/Abstract] OR “neural network*” [Title/Abstract] OR “artificial neural network*” [Title/Abstract] OR “convolutional neural network*” [Title/Abstract] OR “recurrent neural network*” [Title/Abstract] OR “transformer*” [Title/Abstract] OR “foundation model*” [Title/Abstract] OR “large language model*” [Title/Abstract] OR “generative artificial intelligence” [Title/Abstract] OR “generative AI” [Title/Abstract] OR “generative model*” [Title/Abstract] OR “reinforcement learning” [Title/Abstract] OR “supervised learning” [Title/Abstract] OR “unsupervised learning” [Title/Abstract] OR “self-supervised learning” [Title/Abstract] OR “representation learning” [Title/Abstract] OR “computer vision” [Title/Abstract] OR “natural language processing” [Title/Abstract] OR “computational intelligence” [Title/Abstract] OR “expert system*” [Title/Abstract] OR “pattern recognition” [Title/Abstract])
2	Emergence of bioengineering—identifies records explicitly referring to bioengineering in tiab.	“bioengineering” [Title/Abstract]
3	Emergence of AI as an explicit concept—identifies records explicitly using the term artificial intelligence in tiab.	“artificial intelligence” [Title/Abstract]
4	Bioengineering–AI-related convergence—identifies bioengineering records using the broader set of AI-related terms and approaches in tiab.	(“bioengineering” [Title/Abstract] AND (“artificial intelligence” [Title/Abstract] OR “AI” [Title/Abstract] OR “machine learning” [Title/Abstract] OR “deep learning” [Title/Abstract] OR “neural network*” [Title/Abstract] OR “artificial neural network*” [Title/Abstract] OR “convolutional neural network*” [Title/Abstract] OR “recurrent neural network*” [Title/Abstract] OR “transformer*” [Title/Abstract] OR “foundation model*” [Title/Abstract] OR “large language model*” [Title/Abstract] OR “generative artificial intelligence” [Title/Abstract] OR “generative AI” [Title/Abstract] OR “generative model*” [Title/Abstract] OR “reinforcement learning” [Title/Abstract] OR “supervised learning” [Title/Abstract] OR “unsupervised learning” [Title/Abstract] OR “self-supervised learning” [Title/Abstract] OR “representation learning” [Title/Abstract] OR “computer vision” [Title/Abstract] OR “natural language processing” [Title/Abstract] OR “computational intelligence” [Title/Abstract] OR “expert system*” [Title/Abstract] OR “pattern recognition” [Title/Abstract]))
5	Explicit bioengineering–AI convergence—identifies records in which bioengineering and the explicit term artificial intelligence in tiab occur together.	(“bioengineering” [Title/Abstract] AND “artificial intelligence” [Title/Abstract])
